# Extracellular Vesicles as Delivery Vehicles of Specific Cellular Cargo

**DOI:** 10.3390/cells9071601

**Published:** 2020-07-02

**Authors:** Bilal Mir, Claudia Goettsch

**Affiliations:** Department of Internal Medicine I, Cardiology, Medical Faculty, RWTH Aachen University, 52074 Aachen, Germany; bmir@ukaachen.de

**Keywords:** extracellular vesicles, cargo loading

## Abstract

Extracellular vesicles (EVs) mediate cell-to-cell communication via the transfer of biomolecules locally and systemically between organs. It has been elucidated that the specific EV cargo load is fundamental for cellular response upon EV delivery. Therefore, revealing the specific molecular machinery that functionally regulates the precise EV cargo intracellularly is of importance in understanding the role of EVs in physiology and pathophysiology and conveying therapeutic use. The purpose of this review is to summarize recent findings on the general rules, as well as specific modulator motifs governing EV cargo loading. Finally, we address available information on potential therapeutic strategies to alter cargo loading.

## 1. Introduction

Communication between cells is essential to a biological system. Cell-derived signals, via direct cell-to-cell interaction or cell signaling molecules, influence recipient cells’ energetics, biosynthesis and even survival. The transfer of information by extracellular vesicles (EVs) emerges as an additional platform for delivering biomolecules and signals to cells of the surrounding tissues. The presence of EVs in the human body was detected several decades ago, but their sphere of action has not been wholly elucidated until today. The term “extracellular vesicles” describes a heterogeneous class of cell-derived vesicles of different origins, which decelerates the progress of identifying their nature. The originating cell type seems to impact the composition of EVs. Furthermore, several intracellular compartments are involved in sorting and formation processes, which causes additional diversity.

Growing interest in EV biology emerges from results that implicate a crucial role in the progression of several diseases. Interestingly, EVs are a double-edged sword, given their dual role throughout the body in physiological and pathological conditions (reviewed in Mc Gough et al. 2016) [1]. For example, they contribute to the immune response against pathogens or tumor cells, by transferring antigen-loaded major histocompatibility complex II (MHC II) between immune cells [2,3]. In contrast, they are utilized by several viruses for spreading and survival strategy (reviewed in [4]). They can be a carrier of epidermal growth factor (EGF) and its receptor, resulting in improved wound healing after injuries [5], but these EVs also stimulate tumor growth [6]. In addition, cancerous cells benefit from the RNA transport function of EVs, which causes reprogramming and tumor expansion in surrounding cells [7]. In neurodegenerative diseases like Alzheimer’s disease, EVs are linked to the spreading of misfolded proteins [8]. Conversely, by supporting communication between oligodendrocytes and neurons, they are beneficial for the integrity of the central nervous system, [9]. This interaction is essential for neuronal survival. EVs are reported as tools of communication between bone cells, and seem to be involved in the regulation of osteoclast and osteoblast balance by RNA transfer [10]. Yet, they can also initiate microcalcification in vascular or valvular tissue [11], which can result in myocardial infarction or calcific aortic valve disease. These aspects combine to make EVs a topic of huge interest for current medical research. Further investigations of EV biology could provide the opportunity to develop new therapeutic strategies that may contribute to overcoming diverse diseases. 

This review provides an overview of the current state of knowledge of EV subtypes and their life cycles. To understand how EVs function, it is crucial to investigate the cargo sorting process, the EV trafficking and the release mechanisms as far as the uptake by recipient cells. We analyze how an altered cargo influences disease progression, particularly in the context of cardiovascular calcification. We also provide an overview of molecules to alter EV release, which could harbor a potential therapeutic strategy.

## 2. Heterogeneity of Extracellular Vesicles (EVs)

EVs are a heterogeneous group, with still no clear characterization of the particular subtypes. Defined criteria for distinguishing EV subtypes are still lacking, with numerous terms in use to describe EVs—exosomes, microvesicles, oncosomes, prostasomes, argosomes, membrane particles, are some of the many names to appear in different publications (reviewed in [12]). At the first annual meeting of the “International Society for Extracellular Vesicles” (ISEV) in 2012, researchers broached the difficulty of a uniform nomenclature, but could not reach a consensus. Traditionally classified into so-called exosomes, microparticles, and apoptotic bodies, only the expression “extracellular vesicles” was defined as a general term for all naturally released cellular vesicles with a lipid bilayer and without a nucleus and thus replication [13,14]. 

Exosomes are formed by the budding inward of the lumen of endosomes/multivesicular bodies (MVBs) as intraluminal vesicles (ILVs). MVBs are designated to fuse with the cell membrane to release their ILVs to the extracellular space, or can fuse with lysosomes for subsequent degradation. Originally, exosomes were described to reach a size ranging from 0.03 to 0.15 µm in diameter, but recently a size up to 0.25 µm was reported [15]. Tetraspanins (cluster of differentiation (CD) 9, CD63, CD81), the tumor susceptibility gene 101 (TSG101) and syntenin-1 are suggested as specific markers for EVs of endosomal origin [16], although this remains under debate. Additionally, small (<50 nm) extracellular non-membranous nanoparticles named exomeres, which can easily be mistaken as exosomes, have been described recently [17]. They contain metabolic enzymes and are enriched in Argonaute (Ago) proteins [18].

Microparticles, also known as “microvesicles”, “shedding vesicles”, or “ectosomes”, vary in size from 0.1 to 1 µm in diameter. Unlike exosomes, they are characterized by budding directly from the cell’s plasma membrane following phospholipid rearrangement between the inner and outer leaflet [19]. This differing origin compared to exosomes prompted the assumption that another functionality and further effects may be involved. CD29, CD44 [20], C1q and the yeast protein TyA are documented as specific microparticle markers [21]. Recently, Jeppesen et al. revealed Annexin A1 to be expressed more frequently in larger vesicles without tetraspanin expression [22]. The release of both EV subtypes is enhanced by increased intracellular Ca^2+^, given that calcium-dependent enzymes are involved in the formation process [23].

Apoptotic bodies are built by blebbing from the cell membrane during apoptotic disassembly processes [24]. These reach sizes from 1 to 5 µm [25] and present another option to transfer molecules and signals to surrounding cells and could thus prevent further disease progression. Annexin V, which is a marker for apoptosis, is also enriched in apoptotic bodies [26]. 

The classification of EVs into microparticles and exosomes was challenged since vesicles with a diameter in the supposed range of microparticles, but with exosomal markers, were detected [12]. This review uses the terms “exosome” and “microparticle” to describe only the findings that can be related to the specific origin of EVs in accordance with the guidelines of the ISEV. Recent publications distinguish large EVs (lEVs) gathered as an ultracentrifugation pellet at 15,000× *g* speed, from small EVs (sEVs) that can be detected in 120,000× *g* pellets [22]. Typical common EV markers are heat shock proteins (HSP70, HSC70, HSP90) and flotillin-1 [16]. EVs can be isolated by several methods (reviewed in [27,28,29]) like differential centrifugation, density gradient separation, precipitation-based isolation or size-exclusion chromatography. Flow cytometry can be used to characterize only larger EVs, or by using antibodies which are bound to larger beads [30,31,32]. 

Various pathways within the cell influence EV composition and may generate additional EV subpopulations. Furthermore, the EV membrane and cargo seem to differ based on the originating cell type [33]. EV research is not very transparent, with many publications from different research areas. This mass of information has spawned multiple online databases—*Vesiclepedia* (www.microvesicles.org), *EVpedia* (www.evpedia.info) and *ExoCarta* (www.exocarta.org) provide information about protein, lipid and nucleic acid composition in EVs, while *EV-Track* is a website which offers the possibility to exchange information, share experience or seek advice relating to EV research.

## 3. Mechanism of Cargo Sorting into EVs 

EVs can contain a broad spectrum of biomolecules, as described in the following section. For cargo sorting into EVs, different sorting pathways have been described in past decades. Table 1 provides an overview of these mechanisms, most of which are somehow interlinked.

### 3.1. *Pathways of Protein Sorting*

The functionality and destination of EVs differ due to a variation of loaded components, which also modify their membrane composition. Alterations in EV cargo demonstrate influence on disease progression; therefore, cellular components and mechanisms determining the loading process need to be elucidated. The ESCRT (Endosomal Sorting Complex Responsible for Transport) machinery was identified in the context of sorting ubiquitinated proteins into vesicles [34]. While possibly the best examined pathway of EV cargo sorting, it has been known for years that EV formation does not rely on one specific mechanism and alternative pathways exist. 

The ESCRT machinery contains four multi-protein complexes (ESCRT-0/-I/-II/-III) and additional accessory proteins (reviewed in [57]). These can be subdivided into early acting complexes (ESCRT-0/-I/-II)—mainly involved in ubiquitinated cargo sorting—and late acting components (ESCRT-III and vacuolar protein sorting 4 (Vps4)), which terminate EV formation and budding (reviewed in [58]). The early acting ESCRT complexes recruit each other and contain specific ubiquitin-binding domains (UBDs). Studies suggest that ESCRT-0 self-associates at the membrane of endosomes [59] by interacting with its subunit hepatocyte growth factor-regulated tyrosine kinase substrate (Hrs with FYVE-domain) and the phospholipid phosphatidylinositol 3-phosphate (PI3P), which is abundant in the early endosomal membrane [60]. Then, the Hrs compartment interacts with the tumor susceptibility gene 101 (TSG101), which is part of the ESCRT-I protein complex [61]. This complex leads to the assembly of ESCRT-II through Vps28 (ESCRT-I)–Vps36 (ESCRT-II) interaction. Both ESCRT-I and -II contain UBDs for protein recruitment. Several ESCRT proteins can oligomerize and thereby achieve a high avidity, given that they reveal only a modest affinity as monomers [62]. At late stage, the ESCRT-III complex is recruited and activated by Vps25 (ESCRT-II)–Vps20 (ESCRT-III) interaction [63]. ESCRT-III plays a crucial role in EV formation by initiating membrane deformation and inward budding. Its filaments polymerize and form a spiral-like belt, enwrapping the vesicle (reviewed in [58]). ATPase Vps4 is involved in the disassembly of the ESCRT-III complex. Until now, the mechanism behind the formation has not been completely understood. It is known, that the ESCRT machinery is recycled before the budding process is completed [64], and that the cargo loses its ubiquitin-tag prior to vesicle scission via the de-ubiquitinating enzyme-associated molecule with the SH3 domain of STAM (AMSH) [65]. 

In this ESCRT-conducted process, additional interacting proteins are described that take part in EV cargo loading. Neural precursor cell expressed developmentally down-regulated protein 4 (Nedd4) family-interacting protein 1 (Ndfip1) is an endosomal adaptor protein detected in EVs. Besides loading of Nedd4 family proteins into EVs, it can further recruit ubiquitinated proteins labelled with a WW-tag [39,40]. Another important ESCRT-accessory protein is ALG-2-interacting protein X (Alix), which is recruited by charged multivesicular body protein 4a (CHMP4) [66], a subunit of ESCRT-III. This binding leads to a stabilization of the complex. In addition to the ESCRT-I component TSG101, Alix is one of the most abundant proteins in exosomes and widely used as a marker. Alongside its widespread involvement in membrane remodeling processes, Alix can act like an adaptor protein that recruits cargo into developing EVs, in an ubiquitin-independent manner [37,67]. This recruitment was described for G-protein coupled receptor protease activated receptor 1 (PAR1) [37] and the purinergic receptor P2Y1 [67], which are both transferred to the MVB membrane by recognition of their YPX_3_L-motif and the transferrin receptor [68]. Alix is also involved in miRNA recruitment by interaction with the protein complex Argonaute 2 (Ago2) [49]. In addition, it enriches lysobisphosphatidic acid (LBPA) within the prospective EV membrane [69], which is involved in the membrane deformation process, and was suggested as initiator of an additional recruitment pathway by interacting with syntenin, the cytoplasmic adaptor protein. Syntenin binds syndecan and other proteins via their PDZ domain [70]. The Alix-syntenin-syndecan complex, which was described to control around 50% of vesicles in MCF-7 cells [71], can sort specific cargo into EVs. In particular, the syndecan domain heparan sulfate seems to be involved in the sorting and formation process which is cleaved and activated by the modulator-enzyme heparanase [38]. 

Depletion of all four ESCRT-components does not completely prevent ILV formation within MVBs [72], suggesting the existence of ESCRT-independent loading processes. One of the alternative pathways is tetraspanin-dependent. For example, CD63 stabilizes the pre-melanonsome protein (PMEL) in vesicles during melanogenesis [43]. CD9 interacts with the metalloproteinase CD10 [42] and causes an enhanced EV release. It has been noticed that ESCRT-independent vesicles are smaller than ESCRT-dependent, given that depletion of CD63 causes only a decrease of smaller vesicles (<40 nm) [73].

Posttranslational modification by ubiquitin-like proteins (UBLs) seems to be another mechanism to influence EV release and protein recruitment. Interferon-stimulated gene 15 (ISG15) is an UBL protein, inducible by interferons (IFN) [35]. This ISGylation causes the accumulation and degradation of TSG101, resulting in impaired exosome secretion and potentially, subsequent alterations in EV cargo. In addition, UBL3-modification is involved in the protein sorting process of smaller EVs [36].

Less is known about the cargo loading mechanisms of EVs budding directly from the cell membrane. It has long been assumed that the composition of microparticles reflects the cell of origin and the loading process is just passive. However, components of the ESCRT machinery also appear to be involved in the protein sorting of this EV type, although they are directly released and are not intended for lysosomal degradation. The ESCRT-associated ATPase Vps4, as well as TSG101, are reported to play an important role in protein recruitment here [44]. Their interaction with Arrestin Domain-Containing Protein 1 (ARRDC1), which is bound to the plasma membrane, induces relocation of TSG101 via ubiquitin E2 variant (UEV)-motif recognition from the endosomal membrane [44]. The released microparticles are ARRDC1^+^, TSG101^+^, but lack tetraspanins [22].

### 3.2. The Role of Lipids in EV Formation

EVs consist of a lipid bi-layer, given their origin from the plasma or endosomal membrane. Specific lipids are enriched in EVs and may contribute to the sorting and formation process. The membrane of microparticles does not show significant differences in its lipid composition compared to the originating cell membrane [61]. In contrast, the membrane of exosomes is enriched in sphingolipids, glycerophospholipids, ceramide, and cholesterol [74]. The cholesterol content of the endosomal membrane may influence the fate of the arising EVs. While exosomes originating from cholesterol-rich MVBs are determined for secretion, a low cholesterol level directs MVBs to lysosomal degradation [57,75]. EV membrane analysis by Harada et al. revealed at least three different membrane types: low density detergent-insoluble membranes, detergent soluble membrane, and the flotillin-1 enriched high density detergent-insoluble membranes. They identified proteolysis of A disintegrin and metalloproteinase domain-containing protein 10 (Adam 10) and hepatocyte growth factor receptor Met as possible triggers for membrane type switching and thus, alterations in the EV sorting process [76]. Specific membrane domains in the MVB membrane, enriched in cholesterol, sphingolipids and glycosylphosphatidylinositol (GPI)-anchored proteins, are called lipid rafts. MHC II, αB-crystallin or flotillin-1 are examples of raft-associated proteins [45,46] recruited by lipid raft interaction and present in EVs. Furthermore, proteolipid protein (PLP) is abundant in oligodendroglial precursor cell-derived EVs [74] and may contribute to the membrane structure. PLP-containing EVs revealed a similar lipid composition to lipid rafts. Additionally, the release of PLP-enriched EVs is not impaired after silencing of ESCRT-associated proteins [74], suggesting that PLP is part of an ESCRT-independent ILV-forming machinery. 

The neutral sphingomyelinase 2 (n-SMase 2) plays a key role in EV formation, since its inhibition effectively reduces EV release in general, as well as specifically PLP-enriched EVs [74]. The n-SMase 2 converts sphingomyelin in ceramide [1], which is a membrane lipid that deforms membranes, thereby initiating the inward budding process. Its further metabolization into sphingosine-1-phosphate and its receptor is associated with an advanced maturation process and an ESCRT-independent cargo sorting [41]. This involves for example tetraspannin, the transferrin receptor and CD63, which itself is described further on here as a recruiter. The inhibition of phospholipase D2 also decreases EV secretion [77]. It converts lyso-phosphatidic acid into phosphatidic acid, a cone-shaped lipid, involved in the budding process of endosomes and, together with the small GTPase ADP ribosylation factor 6 (ARF6), regulates syntenin and probably the subsequent cargo loading pathway [78].

### 3.3. Transfer of RNA between Cells

It has been several years since any RNA species were identified as EV cargo, but the mechanisms behind the sorting have yet to be fully elucidated. Directed sorting is assumed, due to publications demonstrating differences in microRNA (miRNA) composition between EVs and their cell of origin [50,79]. Besides messenger RNA (mRNA) and miRNA, additional non-coding RNA types like ribosomal RNA (rRNA), transfer RNA (tRNA), Y-RNA and large intergenic noncoding RNA (lincRNA), were found enriched in EVs [22,80]. Not only are recipient cells able to take up these RNAs, but moreover, an adjacent functionality has been proven [81]. 

In human liver stem-like cells the adaptor protein Alix was identified to control EV RNA loading [49]. Alix recruits the RNA-binding protein Ago2 to the endosomal membrane, resulting in miRNA binding and subsequent packaging into EVs. Ago2 is part of the RNA-induced silencing complex (RISC). Both it and the endoribonuclease Dicer are RNA-processing enzymes which have been detected within EVs [82]. Depletion of ESCRT components revealed a decrease of miRNA-mediated gene silencing, changing the image of EVs as bare vehicles to RNA regulation sites [83]. Recent work attributes involvement in miRNA sorting to the Y-Box Protein 1 (YBX1) [51]. Sorting activities were observed in vitro; the explicit mechanism merits further investigation. Additional candidates for miRNA loading are Kirsten Rat Sarcoma (KRAS) [50] and ELAV-like protein 1/human antigen R (HuR) [52].

A short sequence, termed EXOmotif, was detected in miRNAs that are designated as EV cargo [47]. The ubiquitously expressed heterogenous nuclear ribonucleoprotein A2B1 (hnRNPA2B1) binds to an RNA transport signal (RTS) at the 3′ untranslated region (UTR) of the miRNA, containing this motif. This interaction relocates the miRNAs to the EV formation site [47]. Furthermore, hnRNPA2B1 co-localizes with ceramide, the product of the n-SMase [47]. The observation, that n-SMase inhibition caused a miRNA reduction in EVs [84] supports involvement of these factors in the EV miRNA sorting process. Recent evidence suggests that post-translational modifications enable miRNA binding [47] and probably other RNA species [61]. In EVs, sumoylated (SUMO = small ubiqiutin-related modifier) hnRNPA2B1 controls miRNA-binding and may also trigger the sorting [47]. An analog mechanism was reported for the RNA-binding protein Synaptotagmin Binding Cytoplasmic RNA Interacting Protein (SYNCRIP) in hepatocytes [48], which interacts with an EXO-motif and is also sumoylated. SYNCRIP is involved in several processing steps of mRNA and SYNCRIP deficiency reduced the miRNA content in MVB-derived EVs. 

Microtubule-associated protein 1 light chain 3 β (Lc3b), an autophagy marker, was also detected within EVs. These EVs were described to arise from the fusion of MVBs with histone and dsDNA carrying autophagosomes. Recently, it was demonstrated that Lc3b and the conjugated machinery bind RNA binding proteins, like heterogenous nuclear ribonucleoprotein K (HNRNPK) and scaffold attachment factor B (SAFB), which then regulate the small non-coding RNA cargo within EVs [53].

### 3.4. Transfer of Mitochondrial DNA

While the vesicle transfer of RNAs has been long known, recent work identified a transfer of mitochondrial DNA (mtDNA) by EVs; the whole mitochondrial genome was isolated from blood circulating EVs from breast cancer patients [54]. This horizontal transfer of mtDNA was identified as a tool of cancer cells to augment the metabolic activity of recipient cells. Primarily, this mechanism was discovered in the context of hormonal therapy. Breast cancer cells sustain a dormancy stage after blockage of their oxidative phosphorylation. However, this process was inhibited by the mtDNA transfer of hormonal therapy-resistant cells, followed by metabolic recovery of recipient cells and metastatic progression [54]. Even the loading of complete mitochondria within EVs was reported [85]. Occurring in mesenchymal stem cells for instance, it might be a cellular mechanism to remove depolarized mitochondria and overcome oxidative stress. Mitochondria are relocated to the plasma membrane, where they are shed within EVs containing both ARRDC1 and TSG101. These mitochondria can be taken up by macrophages and have displayed an augmenting effect on the metabolism of these cells [86]. However, this study could not completely exclude the contamination of apoptotic bodies. Recently, Jeppesen et al. demonstrated that double-stranded DNA in general is rather associated with non-vesicular release (exomeres) than with EVs [22]. 

### 3.5. Foreign Molecules

Besides the variety of endogenous molecules detected within EVs, foreign molecules can also undergo cargo sorting. This can be a protective mechanism, for example in case of MHC-II bound antigens, which can trigger an immune response. Many viruses manipulate the endocytic pathway and utilize EV release to infect other cells. EVs and viruses often overlap in size ranges, which complicates investigation of viral molecules within EVs [4]. However, the hypothesis that EVs can be infectious was proven by Longatti et al., who demonstrated that subgenomic replicon cells lacking the production of virion structural proteins, were able to infect Huh7 cells [87]. The full genomic RNA of the Hepatitis C virus was packed into EVs and transferred to a recipient cell. This mechanism was also described for Hepatitis A virus [88]. The loading of genomic RNA is only one example of viral components found in EVs. Furthermore, EVs can harbor viral miRNA [89] and non-coding RNA (Epstein–Barr Virus (EBV)) [90], as well as several proteins, like the latent membrane protein 1 (EBV) [91] or glycoprotein B (herpes simplex virus) [92]. A full list was compiled in the review of Khan et al. [93]. For retroviruses, a trojan exosome hypothesis was published in 2003 and describes how these viruses use the endogenous exosome mechanism of the host cell to form infectious, but mimicked particles [94]. For the human immunodeficiency virus (HIV), it is known that the protein negative regulatory factor (Nef) which is encoded in the lentiviral genome, increases EV release to promote infectivity [95,96]. In addition, Nef is loaded into EVs to improve HIV progression in surrounding cells [97]. It was reported that after infection and reproduction within the cell, HIV predominately bud from the cell membrane [98], which basically makes them microparticles. If, or at which point the virus leaves the endolysosomal system is virus type-dependent. Hepatitis C and A virus use this pathway from clathrin-dependent endocytosis to fuse with late endosomes and finally, propagation via EVs [99]. Other viruses interact only with some EV components, like HIV with the ESCRT-machinery [100]. Influenza A virus interacts with members of the Rab family to facilitate transport to, and fusion with, the plasma membrane [101]. With regard to the severe acute respiratory syndrome coronavirus 2 (SARS-CoV 2) which causes coronavirus disease 2019 (COVID-19), no connection to the EV release pathway has been published thus far. Rather, clinical trials are being performed to examine the beneficial effect of mesenchymal stromal/stem cell (MSC)-derived EVs on COVID-19 disease (ChiCTR2000030484; NCT04276987). MSC-derived EVs are reported to have a therapeutic effect in a broad spectrum of diseases. Beneficial effects in ischemia-reperfusion induced kidney injury [102] and protective properties against myocardial infarction [103]; just two of many examples demonstrating their therapeutic potential. These EVs contain a specific enriched subset of miRNAs [104] and proteins [105], modulating cellular response and triggering tissue repair [106]. In the first attempt to treat patients suffering from SARS-CoV 2-induced acute respiratory distress syndrome, Sengupta et al. applied intravenously a single dose of ExoFlo^TM^, an agent containing MSC-derived EVs to a small cohort of 27 patients and demonstrated restored oxygenation, reduced cytokine storm and reconstitute immunity [107]. These data are under debate from leading scientists in the field [108]. Apart from the non-randomization, non-blinding and small sample size, the transparency of the ExoFlo^TM^ product is missing, with the authors providing only a limited amount of information about the origin, composition and characterization of ExoFlo^TM^ [108].

### 3.6. Mineral Crystals as Cargo of Calcifying EVs

In the cardiovascular (CV) field, EVs have become a central point of interest due to a broad spectrum of systemic, but also pathological effects. They are well suited as biomarkers for several cardiovascular diseases; a detailed biomarker cargo list was compiled by Chong et al. [109]. By way of example, EVs have been shown as proinflammatory mediators after myocardial infarction by triggering cytokine release [110]. A subsequent promotion of angiogenesis in the damaged tissue was also reported [111]. 

**Figure 1:**
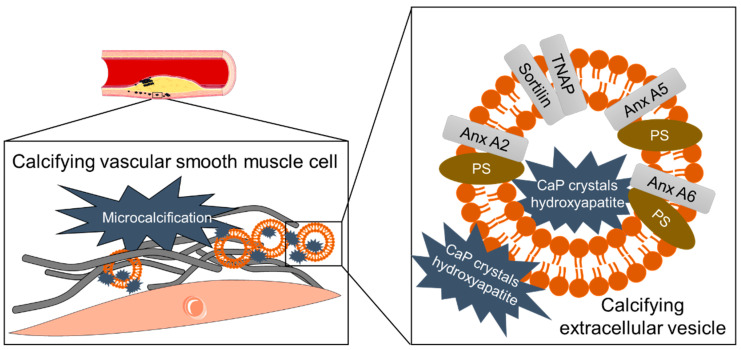
Calcifying extracellular vescilces trigger vascular microcalcification. Microcalcifications are formed within the vessel wall, triggered by released calcifying extracellular vesicles (EVs). These calcifying EVs have a characteristic proteomic and lipid composition. They can be carrier of calcium/phosphate crystals or hydroxyapatite. Anx: Annexin, CaP: Calciumphosphate, PS: Phosphatidylserine; TNAP: Tissue non-specific alkaline phosphatase.

More recently, EVs were identified as calcification initiators in ectopic calcification [11]. Calcifying EVs are released by aberrant macrophages or vascular smooth muscle cells (VSMCs) undergoing osteogenic transformation [112]. Calcifying EVs are of 30–100 nm size [113] and differ in density [114] since they can harbor amorphous calcium phosphate mineral [11], as well as hydroxyapatite [56]. Calcifying VSMCs release EVs prone to calcify and with an altered proteome [114], including an increase of collagen binding proteins like annexins or the tissue non-specific alkaline phosphatase (TNAP), which are linked to calcification (Figure 1) [115]. 

The multiligand sorting receptor sortilin was identified to be crucial for TNAP loading into the calcifying EV membrane [116], which is enriched in phosphatidylserine, with a high affinity to calcium-ions [117]. Annexin A2 is another driver of mineralization, due to its activating effect on TNAP, as well as a reduction of Fetuin-A loading into EVs [118]. In high phosphate conditions also TNAP-negative EVs can nucleate mineralization [119]. This process is linked to an enriched intracellular calcium and simulates chronic kidney disease. The calcification of VSMCs can be blocked by inhibition of the n-SMase 2 that causes a reduction of endosome-derived EVs [55]. Recent studies demonstrated that Imipramine, an acid sphingomyelinase inhibitor, reduces the release of calcifying EVs in bone osteoblasts [120].

## 4. Phosphoinositides Determine the Fate of Intraluminal Vesicles

To identify single cellular components involved in EV formation or cargo loading, it is necessary to fully understand the molecular mechanism behind EV synthesis. Here, it is important to mention that EV formation is not an isolated, independent process; rather, dependent on a well-adjusted balance within the endolysosomal system. EV secretion and autophagy are those cellular processes which maintain cellular homeostasis and both pathways are closely linked. This connection is striking in neurodegenerative disease, where neuronal loss of autophagy causes accumulation of proteins within the cell [121]. The cell then dispose these proteins by enhanced EV release [122], leading to even further propagation [123]. To underline this association, a dysfunction of the EV cargo loading ESCRT-machinery causes autophagosomal accumulation, in turn leading to neurodegeneration [124].

**Figure 2:**
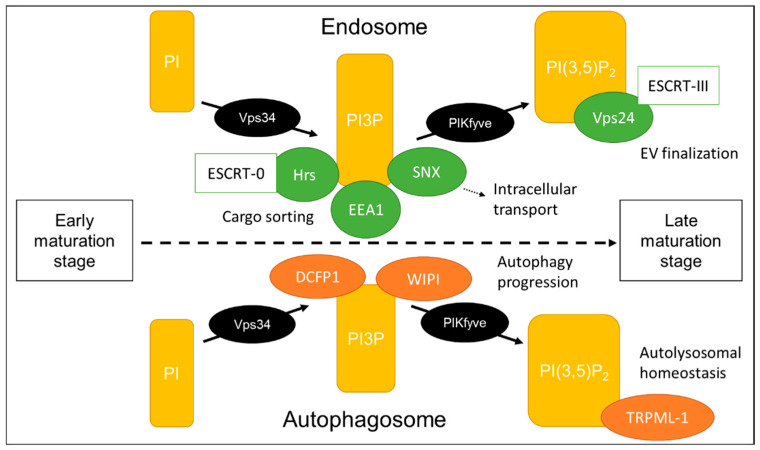
Phosphoinositides conversion defining endolysosomal maturation stage. The conversion of PIs within the endolysosomal compartment membrane allows for the effectors to bind and progress maturation. Although endosomes and autophagosomes share the same central phosphoinositide (PI) conversion, they both have specific effectors important for their specific function. DCFP1: Double FYVE domain-containing protein 1, EEA1: Early endosomal antigen 1, ESCRT: Endosomal sorting complex responsible for transport, Hrs: hepatocyte growth factor-regulated tyrosine kinase substrate, PI: Phosphoinositides, PIKfyve: Phosphatidylinositol-3-phosphate 5-kinase, SNX: Sorting nexin proteins, TRPML-1: Transient receptor potential cation channel 1, Vps: Vacuolar protein sorting, WIPI: WD-repeat protein interacting with PIs.

As constantly processing intracellular compartments, endosomes and autophagosomes, as well as lysosomes, have much in common. Autophagy related proteins (ATGs) are described as taking part in EV formation and release. In the endolysosomal system, the recruitment of membrane proteins is determined by the composition of the compartment membrane, with a central role of phosphoinositides (PIs). The minor presence of PIs (about 10% of phospholipids in eukaryotic cells [125]) belies a major impact on intracellular processes. Their frequency characterizes different compartments, as well as the endolysosomal maturation state (Figure 2). 

Furthermore, PIs are involved in vesicular transport by binding cytoskeletal proteins [126]. PIs are the phosphorylated form of phosphatidylinositol and characterized by an inositol ring, which can be phosphorylated at three different positions (D3, D4, D5). Several phosphatases and kinases are responsible for generating adequate levels of seven possible forms of PI. The most abundant PI is PI4P [127], which is a compartment of the Golgi membrane and involved in trafficking processes [128]. PI3P is less abundant and synthesized by PI3-kinase Vps34. It is enriched in the endolysosomal membranes and recruits other enzymes that are involved in either endosomal trafficking or autophagy [129]. In yeast, unfunctional Vps34 with aberrant PI3P synthesis affected protein sorting and at the same time dysregulated autophagosome formation [130]. The ESCRT-0 associated protein Hrs and the early endosomal antigen 1 (EEA1), both interact with PI3P at the endosomal membrane by their FYVE domain [131], thereby contributing to generation and protein sorting into MVBs. In addition, sorting nexin proteins (SNX) interact with PI3P and enable transport to the trans-Golgi and the plasma membrane [132]. At the autophagosomal membrane, Vps34-complex recruits double FYVE domain-containing protein 1 (DCFP1) and WD-repeat protein interacting with PIs (WIPI), which then trigger autophagy progression. PI3P is converted to PI(3,5)P_2_ or PI5P by phosphatidylinositol-3-phosphate 5-kinase (PIKfyve), a known target to alter EV secretion. PI5P is described as a negative regulator of endosomal maturation and shown to influence protein internalization [133]. PI(3,5)P_2_ is highly expressed on late endosomes, mature autophagosomes and at the lysosomal membrane. Its synthesis is crucial for autophagy progression, since mutations in PIKfyve cause accumulation of autophagosomal marker [134]. Transient receptor potential cation channel 1 (TRPML-1) is another downstream effector of PI(3,5)P_2_ and a regulator of lysosomal size [135]. PI(3,5)P_2_ removes cortactin from actin filaments, thereby regulating compartment movement [136]. It also has an important role in late endosomal protein sorting by binding to Vps24, a component of the ESCRT-III complex [137]. 

Taken together, PIs are fine-tune regulators in the cell and their proportion within the membranes is crucial to defining intracellular compartment identity and maturation stage. One of their diverse functions is the initiation of ILV regulation and cargo loading. They regulate extracellular secretion as well as recycling processes via autophagy, underlining the close connection between these two mechanisms. More and more, PIs are revealing to be an adequate target to influence EV composition and secretion. 

## 5. Inhibition and Promotion of EV Release for Therapeutic Approaches

Successful alterations in EV release can be beneficial in preventing the progression of diverse diseases. For example, in a sepsis mouse model the blockage of ceramide synthesis and subsequent inhibition of EV release, demonstrated a reduction of inflammation and an improved survival rate [138]. The inhibition of EV release in the central nervous system may prevent prion expansion [139] in neurodegenerative diseases. Therefore, EVs offer promising tools for novel treatment strategies. Recent publications demonstrate the successful utilization of EVs as drug-carrying transporters [140,141], to overcome multiple drug resistance in cancer [142] or Parkinson’s [143] disease therapy. In addition, EVs can be loaded with siRNAs, which are efficiently uptaken by recipient cells [144]. Another way to therapeutically load EVs is to intervene in EV biogenesis. One method involves labelling targeted proteins with WW-tag, leading to loading into EVs by Ndfip1 [40]. Of late, efforts have been made to construct EV-mimics for successful drug delivery (reviewed in [46]). An overview of the usage of EVs as therapeutic vehicles was recently reviewed by Melling et al. [145]. 

**Figure 3:**
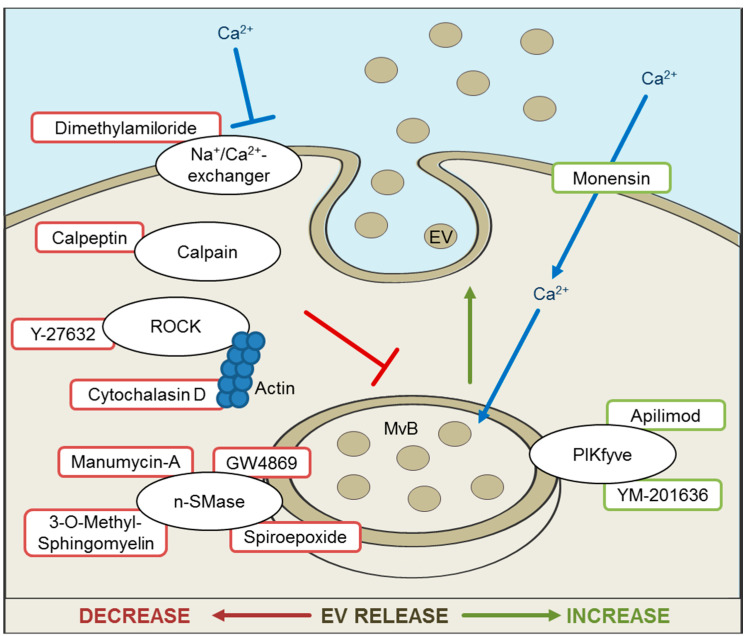
Inhibition and promotion of extracellular vesicle (EV) release. Overview of substances and their targets with enhancing (right side) or inhibitory (left side) effect on EV release. n-SMase: Sphingomyelinase, PIKfyve: Phosphatidylinositol-3-phosphate 5-kinase, ROCK: Rho-associated, coiled-coil containing protein kinase, MvB: multivesicular body.

Besides silencing of proteins involved in EV formation, several compounds were discovered to block or augment EV release (Figure 3). 

One general mechanism of reducing EV release is the interruption of intercellular Ca^2+^ by calcium-chelators. Ionophore monensin is a membrane permeable H^+^/Na^+^-antiporter, which elevates the quantity of intracellular Ca^2+^ [146] and thereby enhances exosomal secretion [147]. Ca^2+^ accumulates within MVBs and causes water influx, leading to extended MVBs. A monesin-like effect has been attributed to Cortactin overexpression. Cortactin is an actin-nucleation factor, involved in endocytosis, cell migration and MVB trafficking [136], and increases MVB-derived EV release. Unlike cortactin overexpression, monesin induces reactive oxygen species (ROS) formation and oxidative stress, prompting apoptosis and changes in EV composition [148]. Further analysis is necessary to examine the effect on alterations of EV cargo. Dimethylamiloride (DMA) blocks H^+^/Na^+^ and Na^+^/Ca^2+^-exchange, suppresses MVB swelling and therefore vesicle release [147]. 

The inhibition of the n-SMase2, via siRNA or pharmacological agents, demonstrated an EV release reduction. GW4869 is a non-competitive inhibitor of the n-SMase 2 and blocks ceramide synthesis, which then suppresses inward budding of MVBs, resulting in a reduction of EV formation. GW4869 was first tested in HEK293 cells [84], but is a prominent tool for inhibiting EV release [138,149]. Other examples for n-SMase inhibitors are 3-O-Methyl-sphingomyelin and Spiroepoxide [55]. The antibiotic Manumycin A is mainly described as a competitive inhibitor of the Ras farnesyltransferase, but is also an irreversible inhibitor of the n-SMase [150]. Both targets are involved in EV synthesis [151], and an inhibition leads to EV release reduction. Yet, the blockage of n-SMase does not inhibit EV release in all cell types, as demonstrated in the prostate cancer cell line PC-3 [152]. It has been suggested that the role of ceramide is cell type-dependent, which could be in line with observed differences in subcellular localization of the n-SMase [152,153]. Tricyclic anti-depressant Imipramine is an inhibitor of acid sphingomyelinase and has demonstrated a reduction of EVs derived from osteoblasts [120].

Another method of inhibiting EV release is to hinder intracellular transport by targeting structural components. Cytochalasin D is a cell permeable mycotoxin which inhibits actin-polymerization and decreases EV release [154]. Y-27632 inhibits Rho-associated, coiled-coil containing protein kinase (ROCK) 1 and 2 by competing with ATP. ROCK contributes to actin formation and membrane deformation. Calpeptin is an inhibitor of calpain, a Ca^2+^-dependent protease involved in many cellular processes [155]. Y-27632, as well as Calpeptin, prevents the formation of EVs budding directly from the cell membrane [156]. 

The inhibition of lipid kinase PIKfyve demonstrated enhanced EV secretion in a prostate cancer cell line [157]. Apilimod (STA-5326) and YM201636 are known PIKfyve inhibitors. PIKfyve phosphorylates PI3P which is abundant in endolysosomal membranes and the conversion causes MVB maturation.

## 6. Conclusions 

Scientific interest in EVs has grown enormously over recent decades, across nearly every biological research field. Not least, the global COVID-19 pandemic demonstrates that besides being possible vehicles for disease spreading, EVs represent a good chance to counter disease. Pooling differently termed vesicles into one big heterogeneous EV group made it possible to combine the knowledge of several separate observations. On the other hand, uncertainty remains as to how to characterize EVs with clearly differing release mechanisms. Furthermore, we need an illumination of the cellular pathways involved in formation, cargo loading, release, and uptake, of which we only have partial knowledge. Here, we provide an overview of observed intracellular structures that are possibly closely linked. We list established strategies to alter EV release, with or without influencing EV cargo. However, when utilizing these effectors, it is necessary to bear in mind the close crosslinking of intracellular processes. By influencing EV release, the autophagy and—in all likelihood—several other cellular processes will also be altered. Inversely, alterations in intracellular membrane composition or autophagy may be a tool to influence secreted EVs too, and thereby, positively affect disease progression. 

## Figures and Tables

**Table 1:** Extracellular vesicle (EV) cargo loading machineries and their reported targets.

Cargo Sorting Machinery	Reported Cargo	References
ESCRT-complex	proteins (ubiquitin-tagged)	[34]
Ubiquitin Binding Proteins (ISG15, UBL3)	proteins (ubiquitin-tagged)	[35,36]
Alix (ESCRT-III associated)	proteins (especially receptors; ubiquitin-independent)	[37]
Alix-Syntenin-Syndecan-complex (Phospholipase D2–ARF6-regulated)	proteins (binding to heparanase sulfate)	[38]
Ndfip1	proteins (Nedd4 family members or WW-tagged)	[39,40]
sphingosine-1-phosphate and receptor	proteins (transferrin receptor, CD63)	[41]
Tetraspannins (CD9, CD63)	proteins (specifically interacting)	[42,43]
ARRDC1–Vps4/TSG101	proteins (microparticle exclusive)	[44]
lipid raft associated sorting	proteins	[45,46]
sumoylated hnRNPA2B1 (ceramide regulated)	miRNA (EXOmotif)	[47]
sumoylated SYNCRIP	miRNA	[48]
Alix–Ago2	miRNA	[49]
KRAS	miRNA	[50]
YBX1	miRNA	[51]
HuR	miRNA	[52]
Lc3b-machinery (associated to RNA binding proteins)	non-coding RNA	[53]
unknown	mtDNA	[54]
unknown	mineral	[55,56]

Ago: Argonaute, ALIX: ALG-2-interacting protein X, ARF6: ADP ribosylation factor 6, ARRDC1: Arrestin domain-containing protein 1, CD: Cluster of differentiation, DNA: Deoxyribonucleic acid, ESCRT: Endosomal sorting complex responsible for transport, hnRNPA2B1: Heterogenous nuclear ribonucleoprotein A2B1, HuR: Human antigen R, ISG: Interferon-stimulated gene, KRAS: Kirsten rat sarcoma, Lc3b: Microtubule-associated protein 1 light chain 3 β, miRNA: micro RNA, mtDNA: mitochondrial DNA, Ndfip1: Nedd4 family-interacting protein 1, Nedd4: Neural precursor cell expressed developmentally down-regulated protein 4, RNA: Ribonucleic acid, SYNCRIP: Synaptotagmin binding cytoplasmic RNA interacting protein, TSG101: Tumor susceptibility gene 101, UBL: Ubiquitin-like protein, Vps: Vacuolar protein sorting, YBX1: Y-box protein 1.
